# An Implantable Intravascular Pressure Sensor for a Ventricular Assist Device

**DOI:** 10.3390/mi7080135

**Published:** 2016-08-08

**Authors:** Luigi Brancato, Grim Keulemans, Tom Verbelen, Bart Meyns, Robert Puers

**Affiliations:** 1 Department of Electrical Engineering, ESAT-MICAS, KU Leuven, Kasteelpark Arenberg 10, 3001 Heverlee, Belgium; robert.puers@kuleuven.be; 2 Department of Cardiovascular Sciences, Experimental Cardiac Surgery, KU Leuven, Herestraat 49, 3000 Leuven, Belgium; tom.verbelen@kuleuven.be (T.V.); bart.meyns@kuleuven.be (B.M.)

**Keywords:** pressure sensor, MEMS, parylene, hemocompatibility, packaging, VAD

## Abstract

The aim of this study is to investigate the intravascular application of a micro-electro-mechanical system (MEMS) pressure sensor to directly measure the hemodynamic characteristics of a ventricular assist device (VAD). A bio- and hemo-compatible packaging strategy is implemented, based on a ceramic thick film process. A commercial sub-millimeter piezoresistive sensor is attached to an alumina substrate, and a double coating of polydimethylsiloxane (PDMS) and parylene-C is applied. The final size of the packaged device is 2.6 mm by 3.6 mm by 1.8 mm. A prototype electronic circuit for conditioning and read-out of the pressure signal is developed, satisfying the VAD-specific requirements of low power consumption (less than 14.5 mW in continuous mode) and small form factor. The packaged sensor has been submitted to extensive in vitro tests. The device displayed a temperature-independent sensitivity (12 μV/V/mmHg) and good in vitro stability when exposed to the continuous flow of saline solution (less than 0.05 mmHg/day drift after 50 h). During in vivo validation, the transducer has been successfully used to record the arterial pressure waveform of a female sheep. A small, intravascular sensor to continuously register the blood pressure at the inflow and the outflow of a VAD is developed and successfully validated in vivo.

## 1. Introduction

Heart failure is the most increasing cause of death in Western countries [1]. Because of the aging population and the difficulty of having a sufficient number of donor organs, device-based therapeutic approaches will play an increasing role in treating patients suffering from severe heart diseases, not only as a bridge to transplantation, but also as a destination therapy [2].

The use of continuous flow heart pumps has become more common with respect to the pulsatile ones because of their smaller size and lighter weight [2,3]. Native ventricles adjust cardiac output depending on preload through the Frank–Starling mechanism. Furthermore, pulsatile pumps can alter filling and ejection times based on preload conditions. Most commercial continuous flow pumps though are operated in a fixed speed setting [4]. If the flow is too high, pressure at the inlet can drop below atmospheric pressure, and a suction event can occur at the inflow cannula. Heart rate, arterial pressure and blood volume, which change with daily activities, affect this unloading point for a fixed pump speed [4]. These events can be detected by monitoring the pressure at the inlet and the outlet of the pump [4,5]. The development of an implantable pressure sensor dedicated to VAD control is the focus of this paper.

An excellent overview of long-term implantable blood pressure sensors is provided by Potkay [6]. As most implantable blood pressure sensors are self-contained devices, they have to provide their own power supply (battery or transcutaneous energy transfer) and data read-out (continuous through wireless link or post-explanation by read-out of the data stored in the implant). Both intra- and extra-arterial pressure sensors have been proposed.

Two Food and Drugs Administration (FDA)-approved intra-arterial sensors are the CardioMEMS™ EndoSensor and the CardioMEMS™ Heart Failure System (St. Jude Medical Inc., Saint Paul, MN, USA) [6,7,8,9]. The sensor principle is based on a passive inductor-capacitor oscillator circuit (LC tank). The capacitive pressure sensor causes a shift in resonance frequency, which can be measured by an external coil. The EndoSensor™ has sensor body dimensions of 30 mm × 5 mm × 1.5 mm and the CardioMEMS™ HF System 15 mm × 3.4 mm × 2 mm. Both sensors have a lifetime greater than three years [6,10].

Systems based on extra-arterial pressure sensors are investigated because of their decreased risk of blood coagulation, drift of the measured pressure output and clotting of the blood stream [6]. The pressure is measured indirectly through the arterial wall: a cuff is wrapped around the artery and is used to sense the expansion and contraction of the vessel. A prototype device of a soft biocompatible rubber cuff (5 mm × 2 mm × 0.1 mm) filled with a low viscosity, biocompatible liquid was developed by Cong et al. [11]. The cuff is wrapped around a mock artery. The overall implant dissipates 300 μW, which is provided by an external adaptive RF powering source. The microsystem was tested in vivo in laboratory rats. Furthermore, Ziaie and Najafi [12] fabricated an extra-arterial blood pressure sensor. The micro-machined silicon capacitive pressure sensor was embedded in a 10 mm × 6.5 mm × 3 mm titanium cuff together with integrated electronics. The devices has a resolution of 0.5 mmHg.

Three studies report the development of a pressure sensor module to monitor the blood pressure at the input or the output of ventricular assist devices [13,14,15]. One solution is to implement the pressure sensor as a flat pressure-sensing diaphragm that is designed to be an integral part of a titanium tube that can be positioned at the inflow or outflow graft of the ventricular assist device [13,14]. Strain gauges are bonded to the thin pressure-sensing diaphragm to detect variations in blood pressure. Due to the need for manual bonding of the gauges, the sensitivity is rather low (1 μV/V/mmHg). The sensor drift over time has been investigated in both cases, although only in vitro, with very different results. The drift in the study of Bullister et al. [13] was very low (1.4–2 mmHg/yr). The sensor from Fritz et al. [14] suffered from a very large drift (−180 mmHg–140 mmHg), which they attributed to the aging of the bond of the strain gauges to the diaphragm. Alternatively, a MEMS pressure sensor (10–20 μV/V/mmHg) can be embedded in the connector of the VAD. In [15], the micro-machined sensor was packaged in a cylindrical capsule filled with silicone oil, which was sealed with a thin segmented polyurethane film (thickness around 50 μm). Only the polyurethane film was in contact with the blood flow. The total package has outer dimensions of 11 by 12 mm. The sensor was tested in vivo with a drift of 24 mmHg over a period of five months [16].

An interesting overview of the current knowledge on hemocompatible coatings for biomedical implants is presented in [17]. Important pathways in the physiological response of the human immune system when a foreign material is immersed in the blood flow include: protein adsorption, thrombin activation, surface coagulation, complement activation, platelet activation and platelet adhesion. Together they define the hemocompatibility of the material. Chemically-inert coatings, such as metal oxides (TiO), nitrides (TiN), silicon carbide and carbon-based inorganic materials, such as hydrogenated amorphous carbon (diamond-like carbon), are used in clinical applications as passive hemocompatible coatings. They act as a corrosion protective layer. Although polymers can more easily be tailored for specific chemical properties to improve hemocompatibility, organic molecules are more susceptible to modifications and hydrolytic degradation compared to inorganic substrates, which display better long-term stability. The hemocompatibility of a particular coating can be improved in three ways [18]: passivation of the surface by long-chained hydrophilic polymers (poly(ethylene glycol), poly(ethylene oxide)) or inert biopolymers (albumin), immobilization of active molecules to interact with blood proteins and cells (i.e., inhibitors to prevent pathways in thrombus formation, for example AT III, thrombomodulin) and the promotion of the growth of endothelial cells. As indicated, the long-term stability of these techniques is still an issue.

Parylene is the commercial name for several polymers of the poly(p-xylylene) family. Due to their chemical vapor deposition (CVD) process, parylenes can be deposited in continuous conformal thin films and are used as moisture and dielectric barriers [19]. In 2002, Weisenberg [20] studied the in vitro hemocompatibility of parylene and other materials used in microelectromechanical systems (Si, SiO${}_{2}$, Si${}_{3}$N${}_{4}$, SU-8) based on platelet adhesion and morphology. Platelet adhesion on Si, Si${}_{3}$N${}_{4}$, SU-8 was significantly greater than platelet adhesion on polyurethane (reference material). Platelet adhesion on parylene and SiO${}_{2}$ was not significantly different from polyurethane.

Although the studies summarized in the previous paragraphs give some indications about the factors influencing the bio- and hemo-compatibility of micro-electro-mechanical system (MEMS) materials and coatings, conclusions drawn depend on many experimental parameters (sterilization technique, process flow, parameters and test procedure). Furthermore, the static fluidic conditions used during these tests do not compare well to the hemodynamic flow inside the blood circulatory system.

This paper presents a feasibility study for the development of a parylene packaged MEMS-based pressure sensor dedicated for close integration with a ventricular assist device. The integration of these sensors in VAD will allow one to monitor the hydrodynamic output of the ventricular assist device and the functioning of the natural heart itself. The hemodynamic data gathered by pressure sensors, together with measurements from other physiological and engineering sensors will enable improved pump control. Additionally, the real-time sensor data will help the physician to make appropriate decisions on the patient’s treatment and to tune the parameters of the VAD for weaning application [3,4,21].

## 2. Materials and Methods

### 2.1. Hemocompatible Packaging of MEMS Pressure Sensor

The implantable sensors were implemented using a commercially-available absolute pressure die from Silicon Microstructure Incorporated (SM5108C). The silicon micro-machined sensor has sub-mm dimensions (650 μm × 650 μm × 650 μm) and a full-scale span of 1550 mmHg. The device consists of a 5-KΩ piezoresistive bridge with four active elements on top of a thin square silicon membrane with a side length of 250 μm. A cavity in the silicon layer, bonded on a glass substrate, provides a sealed reference for this absolute sensor. A similar sensor was administered by Teng et al. [22] in a disposable manometric catheter to measure the intrabolus and peak pressures occurring along the esophageal tract during the swallowing process. Several IC companies supply small land grid array (LGA) packaged manometric pressure sensors with ultra-low power consumption (<100 μW) and a digital readout (I${}^{2}$C or SPI), e.g., Fujikura [23], TDK Electronics [24] and STMicroelectronics [25]. For this application, a bare sensor die was preferred over an already packaged digital pressure sensor. The smallest packaged pressure sensor is from STMicroelectronics and has a dimension of 2 mm × 2 mm × 0.76 mm. Compared to the selected bare silicon die, the LGA packaged sensor would result in a larger unit. Additionally, off-the-shelf sensors contain multiple materials with non-proven biocompatibility.

**Figure 1:**
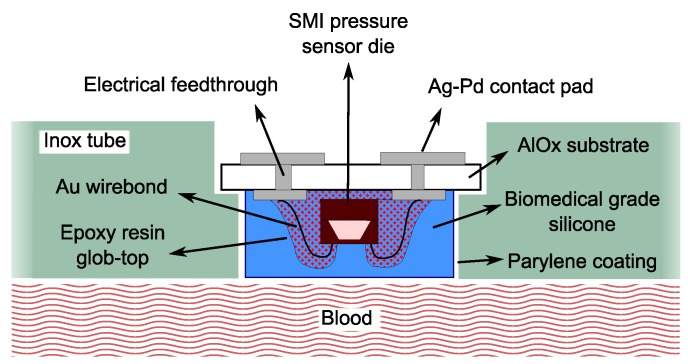
Schematic overview of the sensor embedded in the VAD connector tube.

A schematic view of the device and of the adopted biocompatible packaging strategy is presented in Figure 1. A ceramic thick film process was used. First, small ceramic substrates were produced, with dimensions of 2 mm × 3 mm. Using a screen printing technique, four Ag/Pd contact pads were realized on the top side of each substrate. These contact pads are connected with four other contact pads on the back side of the substrate by electrical feedthroughs. Afterwards, the piezoresistive sensor dies from SMI were glued to the ceramic substrates by medical epoxy under fill (Epoxy Technology 302-3M) and electrically bonded to the Ag/Pd pads by means of gold wire bonds. Carefully avoiding the coverage of the sensitive silicone membrane, the wire bonds were embedded in biocompatible epoxy to confer to the sensor additional resistance against mechanical solicitations.

**Figure 2:**
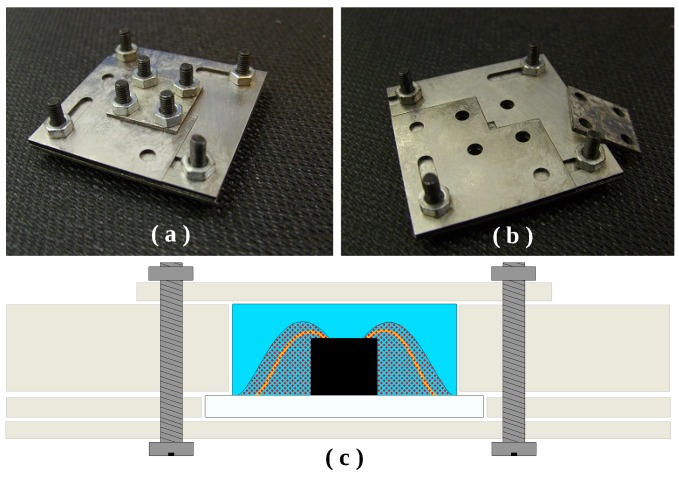
PDMS packaging of the pressure sensor: (**a**) closed mold; (**b**) open mold and (**c**) cross-section of the sensor in the mold during PDMS packaging.

The sensor dies were coated with a 1 mm-thick layer of medical grade polydimethylsiloxane (MED 4211, NuSil, Carpinteria, CA, USA). For the silicone coating procedure, a dedicated mold was designed and realized by laser-cutting stainless steel plates. The molding technique was implemented as follows. With the aid of a microscope, the sample was positioned and immobilized in the center of the mold opening. In the meantime, the PDMS base and curing agent were thoroughly mixed, and the mixture was placed in a vacuum chamber for 30 min for the removal of entrapped air bubbles. Afterwards, the silicone was forced in the mold using a small spatula. A second vacuum cycle of 30 min was applied to remove the air bubbles that could be incorporated during the handling of PDMS. The covering plate of the mold was then positioned and secured by means of screws and nuts. The device was placed in the oven and cured at 45 °C for 5 h in a nitrogen-saturated atmosphere. The mold used is depicted in Figure 2, together with a schematic cross-section, clarifying how the sensor is immobilized during the process.

**Figure 3:**
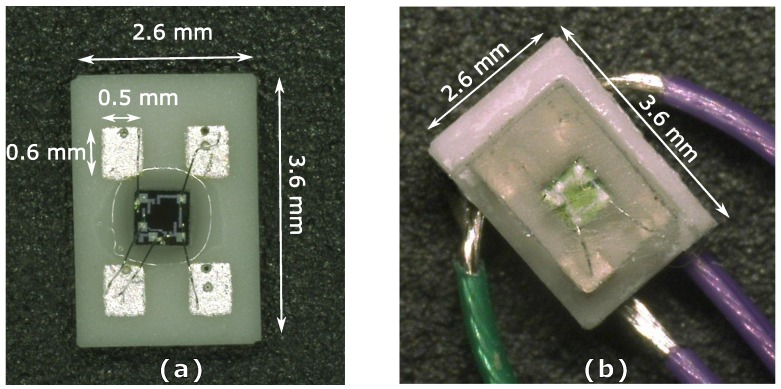
(**a**) Top view of the bare silicon pressure sensor die wire-bonded to the Ag/Pd electrical contact pads on a ceramic substrate; (**b**) silicone-packaged pressure sensor.

After the removal of the device from the mold, lead wires to connect the sensor modules to the readout circuit have been soldered to the Ag/Pd contacts on the back side of the ceramic substrate. The device with dimensions of 2.6 mm × 3.6 mm × 1.8 mm is shown in Figure 3 before and after packaging.

PDMS was selected as a packaging material because it is biocompatible, easy to mold in the desired shape and possesses excellent bulk material properties. This first packaging layer provides mechanical protection to the underlying wire bonds and the delicate silicon membrane. However, because of its relatively high permeability to water vapor (128.33 $\frac{g\times mm}{m^{2}\times day}$ [26]), this polymer alone does not qualify as a reliable barrier against fluid diffusion.

Reliable barrier properties, together with the need for a non-thrombogenic material, are the biggest challenge for devices that are continuously exposed to the blood flow. For this reason, a second uniform coating layer of parylene-C with a thickness of 4 μm was deposited on the device via chemical vapor deposition. Due to its high bulk resistivity, its excellent chemical resistance and its low water vapor permeability (0.08 $\frac{g\times mm}{m^{2}\times day}$ [19]), parylene-C is the material of choice to protect the implant from aggressive body fluids.

When used as a bridge to heart transplantation for patients suffering from severe heart disease, only 55% of the VADs remain implanted for longer than 12 months. However, when the VAD is selected as a destination therapy, the patient survival rate is 48% after 48 months from the implant [2]. Moisture diffusing through the packaging layers during these months could lead to corrosion, short circuits and, consequently, failure of the sensor. The PDMS layer alone would allow for diffusion of up to 39 mg of water per month. The additional parylene-C layer limits the ingress of water to less than 3 mg per month.

**Figure 4:**
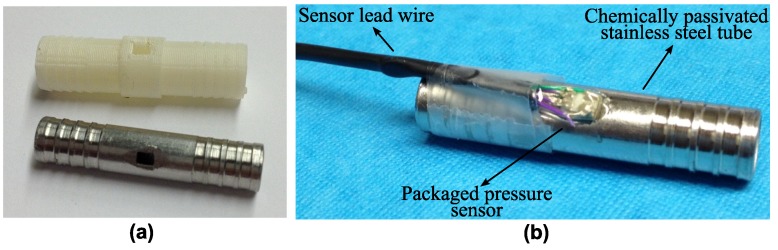
(**a**) Connector tubes used for testing purposes; (**b**) pressure sensor integrated in a stainless steel connector.

Aside from the development of the biocompatible packaging strategy described above, an effort was made to provide a robust mounting of the sensor in the inflow and the outflow connectors of the heart pump. For preliminary functional tests on the packaged device, two dummy versions of the connector were realized. For rapid and low-cost functional testing of the sensor, plastic tubes in ABS were 3D-printed. For all later in vitro and animal tests, machined stainless steel connectors were prepared. The connectors are presented in Figure 4a, whereas Figure 4b shows a sensor integrated in the stainless steel tube.

The packaged device is fixed by means of medical-grade epoxy with the sensitive membrane facing the inner lumen of the tube, towards the blood flow. In order to promote the biocompatibility of the inox part, the alloy was chemically passivated by submerging it in a solution of nitric acid 20% (*v/v*) at 60 °C for 30 min to promote the formation of an oxide layer.

### 2.2. Sensor Readout

**Figure 5:**
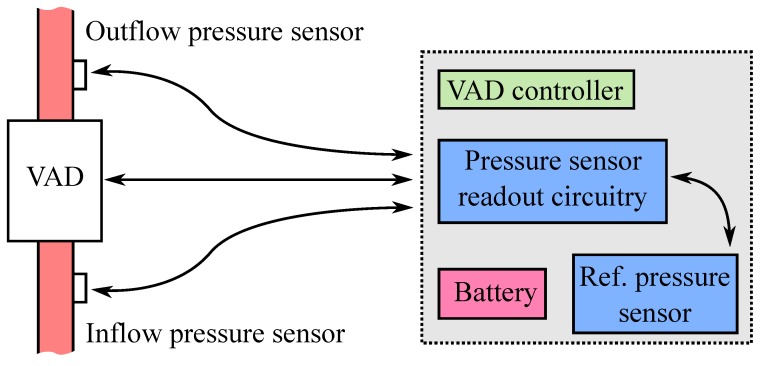
VAD architecture with integrated inflow and outflow pressure sensors and readout.

Figure 5 shows the intended high level integration of the implantable sensors and the interfacing of the sensor readout with the VAD controller. The two sensors, integrated in the input and output cannulae of the ventricular assist device, are connected to the sensor conditioning circuit via a wired connection. The readout board can be considered as an ad hoc unit of the VAD controller, allowing one to take actions based on pressure dynamics. The VAD controller and battery are worn by the patient on a belt or jacket outside the body. Power for the sensors and readout board is provided by the battery of the VAD. Since the implantable sensors are absolute pressure sensors, an extracorporeal pressure sensor is used for compensating for changes in atmospheric pressure.

To investigate the performance of the implantable sensor during in vitro and in vivo tests, a prototype printed circuit board (PCB) was developed. Two sensors, integrated in the input and output cannulae of the ventricular assist device, are attached to the conditioning circuit via wired connections. The devices are powered by a dedicated bridge drive circuit and read out in a constant current mode. This prevents changes in the resistance of the lead wired from affecting the measured pressure value. Besides pressure, also temperature is recorded for calibration purposes. The analogue signals are amplified by an instrumentation amplifier (INA333, Texas Instruments, Dallas, TX, USA), filtered and subsequently converted to the digital domain (ADS1118, Texas Instruments, Dallas, TX, USA). A sampling frequency of 64 Hz is chosen for all signals. The cut-off frequency of four anti-aliasing filters is fixed at 12 Hz. The digital data are read out by an on-board microcontroller (PIC16LF1827, Microchip Technology, Chandler, AZ, USA) over an serial peripheral interface (SPI) bus. As extracorporeal reference pressure sensor, the absolute digital pressure transducer MS5803-01BA from Measurements Specialties, was selected. An on-board universal synchronous-asynchronous receiver-transmitter (USART) to universal serial bus (USB) interface (FT232B, Future Technology Devices International (FTDI), Glasgow, UK) also enables continuous read-out and logging of the sensor output signals on a PC or laptop. The PCB has dimensions of 8 cm by 2 cm. The total power consumption of the board is 15 mW, which is acceptable since the typical power to drive a VAD is several watts.

## 3. Results and Discussion

In order to evaluate the performances of the intravascular pressure sensor and its biocompatible packaging technique, the device was submitted to extensive functional testing. An initial study was performed to investigate how several parameters influence the sensor characteristic. After assessing the long-term stability of the sensor assembly and packaging in vitro, the transducer was submitted to the first in vivo validation.

### 3.1. Influence of the Sensor Packaging on Sensor Sensitivity

**Figure 6:**
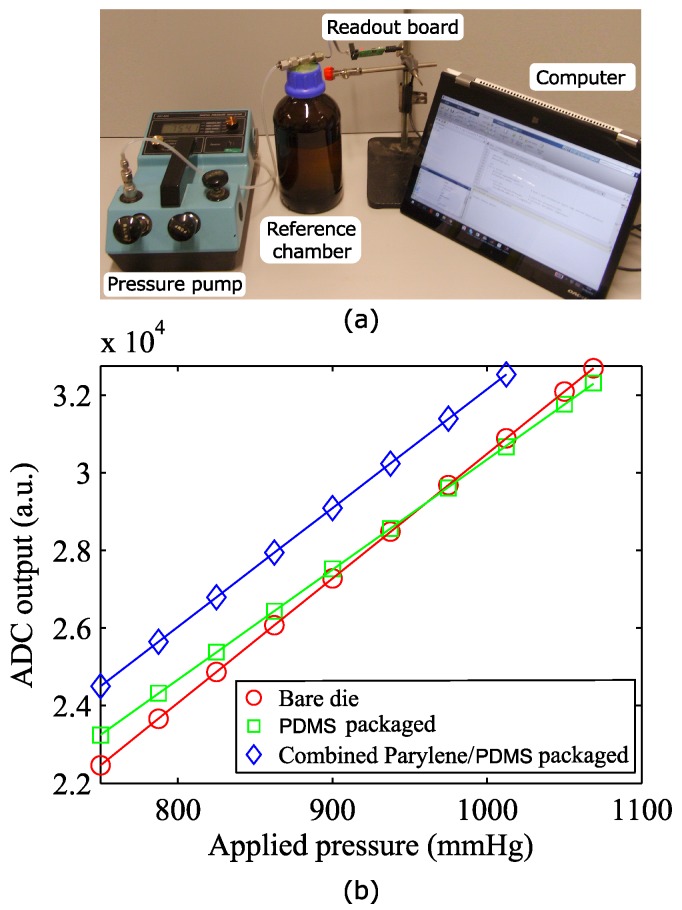
(**a**) Calibration setup; (**b**) plot of the ADC registered value as a function of the absolute applied pressure for: the bare die (red, circle), the PDMS-packaged (green, square) and combined parylene/PDMS-packaged sensor (blue, diamond).

The SM5108C (SMI pressure sensors, Milpitas, CA, USA) is an extremely small piezoresistive pressure sensing chip. Aside for the small dimensions, this device was selected because of its high sensitivity and its strongly linear response in the pressure range of interest. Those characteristics hold true for the bare die. However, the coating layers applied on the sensor and the integration in the VAD connector tube can influence its performance and need to be investigated. The device to be tested was placed in a sealed glass chamber, and different pressure values were applied by a manual pump unit with an internal reference pressure sensor used for calibration purposes (Figure 6a). Absolute pressure values ranging from 750–1100 mmHg were considered, corresponding to a maximum blood pressure of 350 mmHg. The pressure values recorded by the implantable sensor were logged on a calculator using the read-out board developed. Figure 6b demonstrates the linear relation between the digital values recorded by the ADC and the applied pressure for a bare die, a PDMS-coated sensor and a combined parylene-C/PDMS-packaged pressure sensor.

The PDMS packaging causes the device sensitivity to drop by 10%, when compared to the bare die, with an additional offset of 8% of the instrument’s full scale. A further offset shift of 12% full scale (FS) is measured after deposition on the device of the second coating layer of parylene-C. Presumably, this stiff polymeric layer limits the ability of PDMS to relax the stresses in the packaging by deformation, therefore causing a restoration of the original sensitivity, annihilating the negative effect of the PDMS coating.

**Figure 7:**
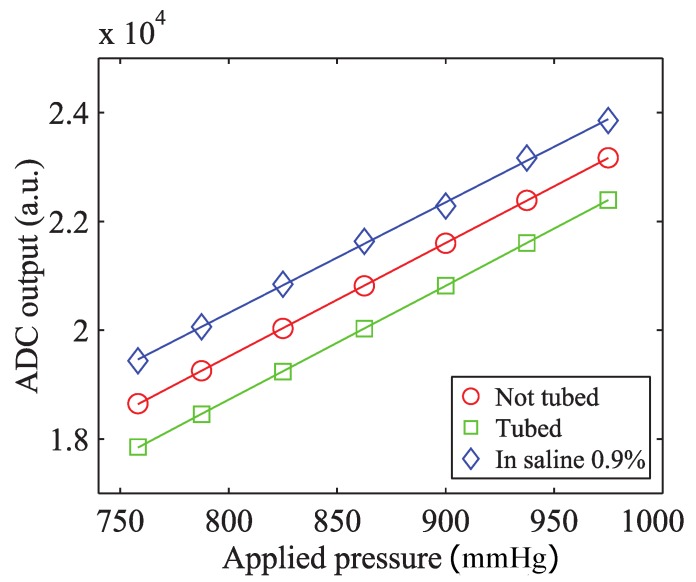
Functional testing of the packaged sensor before (red, circle) and after (green, square) its integration in the connector tube and in saline solution 0.9% (blue, diamond).

To investigate how the fixation in the metal tube affects the output of the device, the same test was performed on a packaged sensor after integration in the stainless steel connector tube. A droplet of medical-grade epoxy (302-3M, Epoxy Technology, Billerica, MA, USA) was used for the fixation of the sensor in its housing. Moreover, in order to test the behavior of the sensor in a wet environment mimicking the conductive properties of the body fluids, the same measurement was performed immersing the tube in saline solution 0.9% at 37 °C. The results are plotted in Figure 7.

The previous experiment demonstrated for all of the three cases examined a strongly linear relationship between the applied pressure and the output of the device. The 8% full scale (FS) negative offset registered after gluing the sensor in the connector tube can be explained by the following consideration. In order to avoid any blood leakage, the device shape is designed to tightly fit into the laser-cut opening in the VAD connector. The insertion of the sensor in the metal tube can induce additional stress in the sensor package determining an offset in the measured value of pressure. The geometrical confinement in the metal tube prevents any relaxation of those stresses over time. The difference between the coefficients of the thermal expansion of the materials used in the package causes an additional 16% FS offset when the device is submerged in saline solution at 37 °C. These changes in the offset, clearly indicate the need for the calibration and compensation of the sensor under operating conditions (tubed, at 37 °C). No significant change in the sensitivity was measured. The sensitivity of the packaged sensor is approximately 12 μV/V/mmHg. This value is comparable to one of the devices proposed by Saito et al. [15,16] and 10-fold higher than the sensitivity of VAD-specific implantable sensors by Bullister et al. [13] and Fritz et al. [14].

### 3.2. Influence of Temperature on Sensor Sensitivity and Offset

To determine how the voltage output by the ADC relates to the actual blood pressure, each transducer should be individually calibrated. The coefficient of sensitivity and the zero offset of the device have to be determined keeping in mind that for a piezoresistive bridge, thermal variations can cause significant changes in these parameters. It is therefore necessary to study the temperature dependence of the sensitivity and offset coefficients, respectively *S*(*T*) and *Off*(*T*). Therefore, the calibrated value of pressure can be calculated using Equation (1).

(1) $$P_{calibrated}=\frac{P_{measured}-\mathrm{Off}\left(T\right)}{S(T)}$$

**Figure 8:**
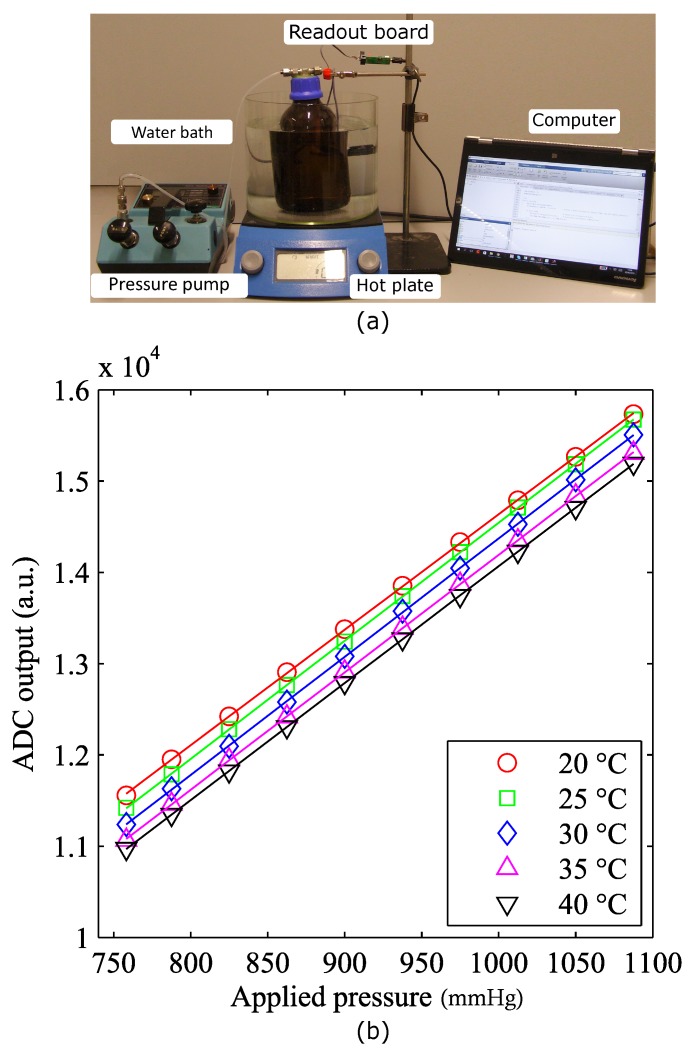
(**a**) Temperature sensitivity measurement setup; (**b**) plot of the measured ADC output as a function of the applied pressure for different temperature values.

The calibration procedure was performed at a fixed temperature on a hotplate (Figure 8a). The sensor being tested was placed in a sealed chamber filled with saline solution and introduced in a water bath. A thermocouple was used to monitor the temperature of the water, while a mechanical stirrer ensured uniform temperature distribution in the bath. The output of the sensor was then measured for increasing values of pressure. This measurement was repeated for different temperatures ranging from 20 to 40 °C. In Figure 8b, the voltage obtained by the ADC was plotted as a function of the applied pressure for five different temperature values.

**Figure 9:**
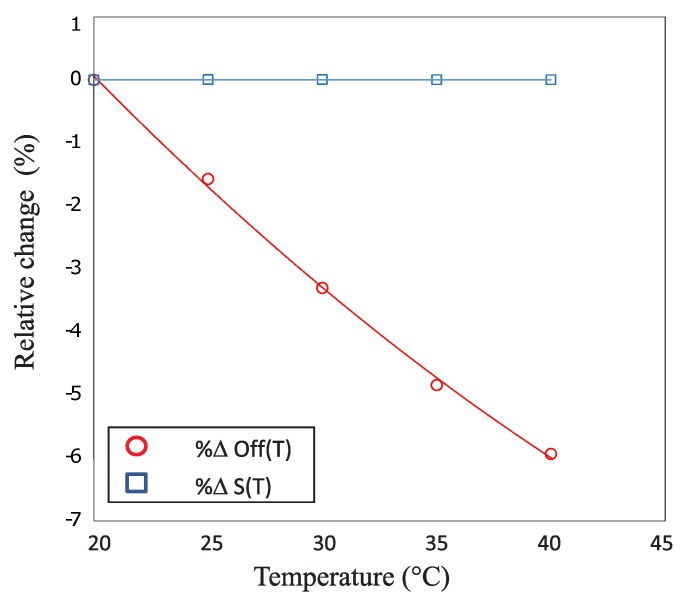
Offset (red, circle) and sensitivity (blue, square) relative changes as a function of temperature.

When heating the saline solution from room temperature up to 40 °C, an increasing zero offset was registered. This effect was most probably due to the dissimilar coefficients of thermal expansion of the different materials causing structural stress to appear at the interface between the multiple coating layers. In Figure 9, the sensitivity and offset are plotted as functions of temperature. Each data point represents the deviation from the reference value measured at 20 °C and is calculated as the percentage of the full scale of the device (Equations (2) and (3)). The trend lines fitting the two datasets are also displayed on the same graph.

(2) $$\%\Delta \mathrm{Off}=100\times \frac{\mathrm{Off}\left(T\right)-{\mathrm{Off}}_{0}}{FS}$$

(3) $$\%\Delta S=100\times \frac{S\left(T\right)-S_{0}}{FS}$$

The sensitivity of the device, in the temperature range of interest, changes by less than 0.01% FS. Therefore, it can be considered constant and does not require any compensation. The offset, in contrast, drops by 6% FS during the experiment. It exhibits a quadratic dependence on the temperature and therefore requires a second order compensation. These parameters can be calculated from Equations (4) and (5):

(4) $$\mathrm{Off}\left(T\right)≃K_{1}+K_{2}\left(T-T_{0}\right)+K_{3}{\left(T-T_{0}\right)}^{2}$$

(5) $$S\left(T\right)≃S_{0}$$

where *K*${}_{1}$, *K*${}_{2}$ and *K*${}_{3}$ are calibration constants to be obtained for each single sensor. These calculations are performed in the microcontroller on the sensor readout board.

### 3.3. Leakage Test

**Figure 10:**
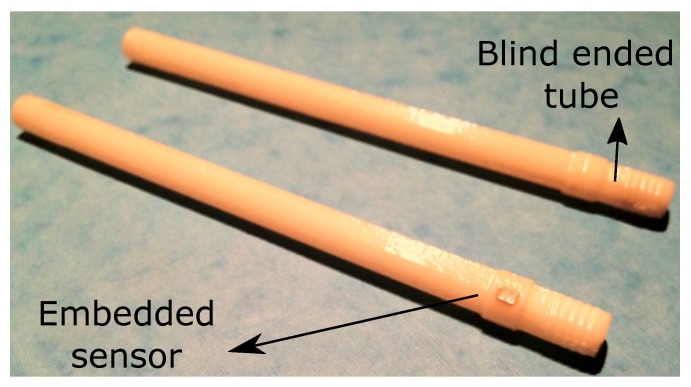
3D printed tube used for physical leakage test.

It is essential, for the sensorized connectors of the heart pump, to prevent any leakage of blood from the pressurized cannulae. Medical-grade epoxy (Epo-Tek 302-3M, Epoxy Technology Inc., Billerica, MA, USA) was used to provide a robust mounting of the sensor in the connectors, while offering a reliable sealing of the opening in the metal tube. To validate the quality of the sealing, a helium leakage test was performed. For this purpose, a dedicated connector tube was realized using a 3D printing technique (Figure 10).

After gluing the sensor, the tube was pressurized with helium, applying a pressure of 1400 mbar by means of a helium gun. The flow of gas through the sealing, measured with a sniffer probe (HLT 572, Pfeiffer Vacuum, Aßlar, Germany), was read out on the screen of the leak detector. The use of a longer connector reduced the influence on the measurement of any helium leakage at the interface between the tube and the helium gun. Prior to the experiment, a layer of insulating varnish was applied on the tubes, and a preliminary measurement was conducted on a blind ended sample, to prove that no helium is leaking through the wall of the tube itself. For all of the measurements, the readout was lower than 10${}^{-8}$ mbar L/s, that is smaller than the minimum resolution of the instrument.

### 3.4. In Vitro Validation of the Implantable Sensor

**Figure 11:**
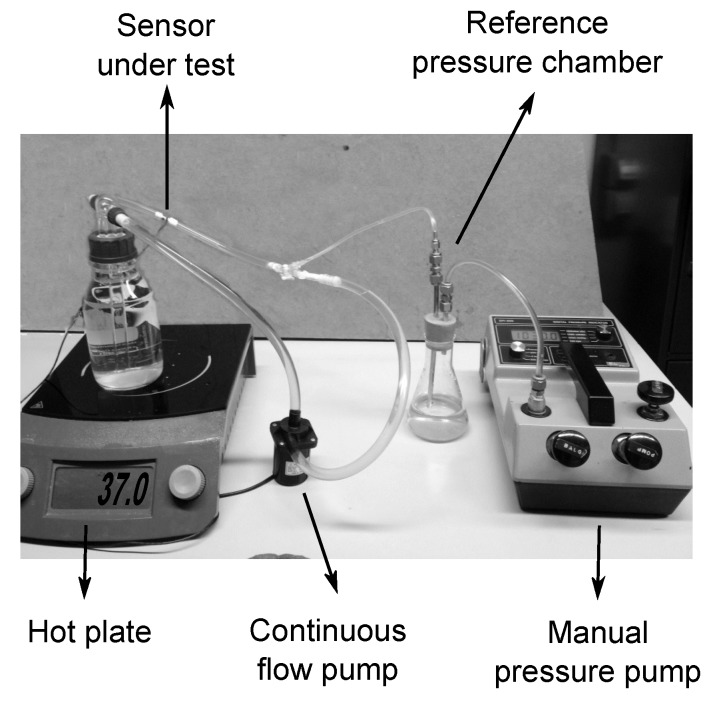
Experimental setup used for in vitro testing.

When exposing a sensor to the blood stream, there are two factors that have to be taken into account. First, the introduction of a foreign body in the blood flow will induce a physiological response that might lead, in the worst case, to the formation of a life threatening blood clot. Second, the exposure of the sensor to an aggressive body fluid, such as human blood, can also be critical for the device itself. Blood components’ deposition on the surface of the device or leakage of material through the packaging could result in modifications of the sensor properties that could lead to changes in the sensitivity and the drift of the baseline of the sensor. The continuous mechanical solicitation of the fluid on the outer packaging layers can also contribute to drastically reducing the lifespan of the device. A dedicated in vitro platform was implemented to assess the stability of the selected bio- and hemo-compatible packaging strategy under flow conditions (Figure 11).

The aforementioned setup was used to study the performance of the sensor during a time span of 15 days. The short shelf life of blood at room temperature made it an unsuitable candidate for this test. Furthermore, the use of preservatives and anticoagulants would influence the natural viscosity of the fluid, eliminating the advantage of using real blood. For these reasons, to artificially recreate the mechanical solicitations that will act on the device once introduced in the circulatory system, the sensor was integrated into a stainless steel connector tube and exposed to a continuous flow of saline solution 0.9% at 37 °C.

**Figure 12:**
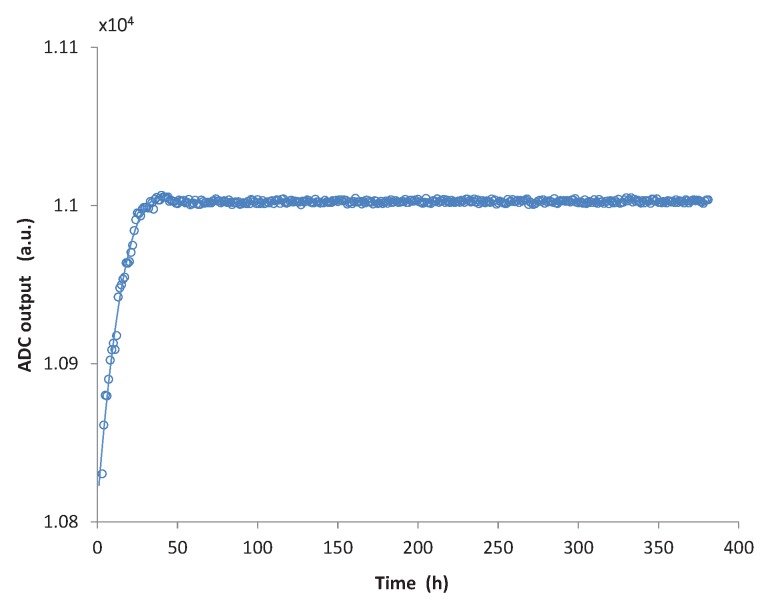
Drift of the sensor during the first fifteen days of in vitro testing.

The saline solution was pumped in a closed loop circuit using a continuous flow pump with a flow rate of 4 L/min, which is an average blood flow rate and falls within the range of most VAD systems [27,28,29]. Additionally, a manual pressure pump was connected to the circuit, through a reference pressure chamber, allowing one to monitor and control the pressure in the closed loop. The output of the sensor was monitored by continuously logging data on a calculator. The acquired data are displayed in Figure 12.

In the graph, the *x* axis displays the hours elapsed from the beginning of the measurement, whereas on the *y* axis, the output of the analogue to digital converter for atmospheric pressure, as measured from the sensor, is reported. Each point represents the calculated average for about an hour of measurement. During the first two days from the beginning of the measurement a significant drift of the baseline of the sensor is registered. During the first 10 h, the drift rate reaches 10 units/h. Considering that the measurement was performed at atmospheric pressure (750 mmHg), this value corresponds to an initial drift of 0.7 mmHg/h. However, this drift rapidly decays to about 0.12 mmHg/h after 20 h and, after 50 h of measurement, the output of the sensor remains stable on values comparable with what was reported by Bullister et al. [13] and Saito et al. [15] and much smaller than the sensor drift measured by Fritz et al. [14].

This drift of the sensor can be attributed to a number of factors, including aging of the PDMS, swelling and thermal stress redistribution at the interface of different materials. These processes can last months or even years at room temperature and below. A “burn-in” of the device by increasing the temperature well above the normal operating point will determine a faster aging of the sensor that will result in the reduction of the initial offset drift. Therefore, additional tests are being performed to evaluate the drift of a packaged device after post-baking it for 2 h at 150 °C in an inert N${}_{2}$ atmosphere.

### 3.5. In Vivo Validation

For a preliminary in vivo validation of the implantable pressure sensor, it was decided to introduce the device in the arterial circulation of a sheep. The goal of this test was to prove the in vivo functionality of the device and to investigate the physiological response induced in the blood by the outer packaging layer of the sensor.

The stainless steel connector presented in Figure 4 was used for this experiment. This part consists of a hollow cylinder with an external diameter of 6 mm and a length of 30 mm. On each end of the connector, four grooves were machined to allow a reliable fixation of the blood vessel on the tube. A hole with the exact dimensions of the packaged sensor was laser-cut on the connector. To improve the material corrosion resistance, and thus its biocompatibility, the tube was chemically passivated submerging it in a solution of nitric acid to promote the formation of an oxide layer. Finally, a completely packaged pressure sensor was glued in the tube using medical-grade epoxy.

**Figure 13:**
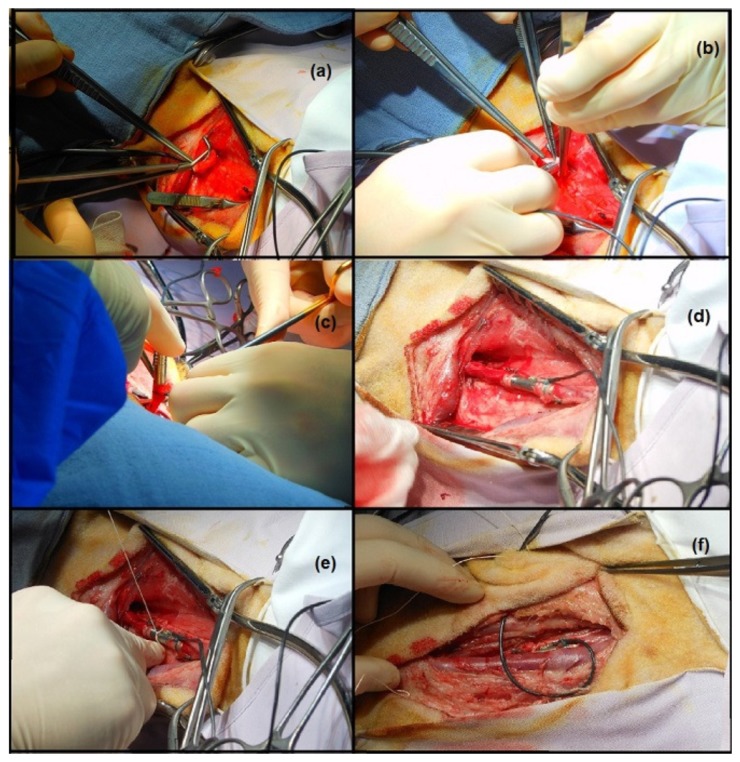
Pressure sensor implant, surgical procedure: (**a**) Carotid artery transection; (**b**–**d**) Insertion of the sensor tube; (**e**) Stitching of the tube on the underlying muscle; (**f**) Subcutaneous tunneling of the sensor’s wires.

This study was approved by the KU Leuven animal ethics committee (P172/2009). The animal received humane care in compliance with the ‘Principles of Laboratory Animal Care’ formulated by the National Society for Medical Research and the ‘Guide for the Care and Use of Laboratory Animals’ prepared by the Institute of Laboratory Animal Resources (National Institutes of Health). One ewe (Swifter-Charolais) of 45 kg, aged 10 months was included. After sedation with intramuscular ketamine 15 mg/kg, anesthesia was induced with isoflurane. The sheep was positioned in a right lateral position. After intubation, the animal was mechanically ventilated with the use of a volume-controlled respirator (Dräger, Cicero, Telford, PA, USA). Anesthesia was maintained with isoflurane (2%–3%) in a gas mixture consisting of 80%–100% oxygen supplemented with room air. The ruminant stomach was decompressed with a 12-bore orogastric tube. Anesthesia was monitored by checking eyelid reflexes and continuous monitoring throughout the study of end tidal CO${}_{2}$ (respiratory volume and frequency were adapted to keep it within the range of 35–45 mmHg), ECG, arterial blood pressure, heart rate and blood O${}_{2}$ saturation. Buprenorphine-hydrochloride 0.3 mg and Meloxicam 0.5 mg/kg IV were used for analgesia. Figure 13 illustrates the critical moments of the operation. A pressure line in the left ear artery served to measure arterial blood pressure. The left carotid artery of the animal was selected as the target location for the implant, due to its dimensions, comparable with the diameter of the tube, and its easy accessibility.

After a length incision at the left side of the neck, the jugular vein, the vagal nerve and the carotid artery were identified and separated. The carotid artery was clamped twice and in between the clamps transected (Figure 13a). The vessel was then fixed on the two edges of the tube using small nylon ropes, (Figure 13b–d), and the tube itself was fixed by means of sutures on the underlying muscle tissue (Figure 13e). Finally, the wires were tunneled under the skin (Figure 13f), and the incision was closed.

**Figure 14:**
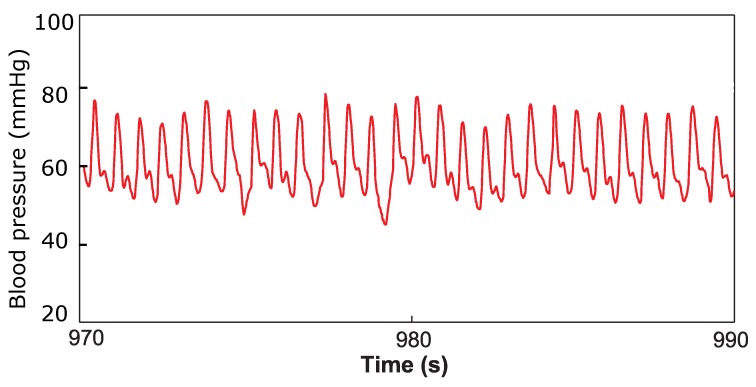
Twenty-second window of the arterial pressure waveform acquired during the in vivo experiment.

Immediately after the implantation, while the animal was still anesthetized, a one-hour measurement was taken to assess the functioning of the device in contact with the blood. The output of the implantable pressure sensor was logged on a computer, and a typical arterial pressure waveform was registered as displayed in Figure 14. The pressure in the left ear, measured by the catheter, was simultaneously recorded as a reference signal. The two signals displayed good correlation, with a correlation coefficient of 0.87. Thereafter, the animal was awakened. The device stayed functional up to 12 h from the beginning of the measurement when, due to the movements of the sheep, damage to the readout electronic and sensor occurred, forcing us to prematurely terminate the experiment.

The preliminary test performed proved the feasibility of successfully measuring arterial pressure signals from a living animal. However, a more extensive in vivo validation is required to assess the stability of the device in the long term: blood components’ deposition on the surface of the sensor or leakage of material through the packaging could result in modifications of the device properties that could lead to sensitivity changes and baseline drift.

## 4. Conclusions

A compact intravascular pressure sensor to monitor the hydrodynamic output of a ventricular assist device was designed and fabricated. The collected sensor data will support physicians in tuning the parameters of the VAD to the needs of the patient. A hemocompatible packaging strategy was implemented, based on a double coating of PDMS and parylene-C, and an electronic circuit for conditioning and read-out of the pressure signal was developed.

The device was first tested in vitro to assess the performance of the proposed packaging technique. It was demonstrated that the successive coating layers do not alter the linear response of the piezoresistive device in the pressure range of interest. The sensitivity of the die was not affected either by the packaging materials or by the integration in its housing connector. However, an offset shift is introduced in the measured pressure value during packaging and needs to be addressed. A calibration, which also allows one to compensate for changes in the temperature during operation, is performed on the microcontroller integrated on the low power read-out board. When exposed in vitro to a continuous flow of saline solution, the device output remained stable for more than 350 h: after the initial 50 h, the sensor displayed a very low drift (less than 0.05 mmHg/day).

To conclude, the functionality of the device was demonstrated by successfully recording the arterial pressure waveform from the carotid artery of a sheep during animal experiments. Nevertheless, a longer and more extensive in vivo validation of the sensor is needed in order to investigate its performance in blood in the long term and to assess the thrombogenicity of the proposed coating. Further animal experiments are being performed implanting two intravascular pressure sensors in the inflow and the outflow cannula of a VAD. The goal of these tests is to prove the possibility to measure the pressure step across the pump for different speed settings. By steadily increasing the speed of the VAD and by monitoring the pressure signals, it will be possible to induce and detect suction events. Surface modification techniques aimed at minimizing platelets deposition on the parylene surface are currently being investigated.

## Abbreviations

The following abbreviations are used in this manuscript:

MEMS: Micro-electro-mechanical system
VAD: Ventricle assist device
PDMS: Polydimethylsiloxane
FDA: Food and Drugs Administration
LC tank: Inductor-capacitor oscillator circuit
RF: Radio frequency
CVD: Chemical vapor deposition
IC: Integrated circuit
LGA: Land grid array
I${}^{2}$C: Inter-integrated circuit
SPI: Serial peripheral interface
ABS: Acrylonitrile butadiene styrene
PCB: Printed circuit board
USART: Universal synchronous-asynchronous receiver-transmitter
USB: Universal serial bus
ADC: Analog to digital converter
FS: Full scale
